# Predicting Universal Healthcare Through Health Financial Management for Sustainable Development in BRICS, GCC, and AUKUS Economic Blocks

**DOI:** 10.3389/frai.2022.887225

**Published:** 2022-04-29

**Authors:** Manoj Kumar M. V., Nanda Kumar Bidare Sastry, Immanuel Azaad Moonesar, Ananth Rao

**Affiliations:** ^1^Department of Information Science and Engineering, Nitte Meenakshi Institute of Technology, Bangalore, India; ^2^Ramaiah Medical College and Hospitals, Bangalore, India; ^3^Health Administration & Policy, Mohammed Bin Rashid School of Government, Dubai, United Arab Emirates; ^4^Dubai Business School, University of Dubai, Dubai, United Arab Emirates

**Keywords:** UHC, healthcare financial management, sustainable development, artificial intelligence, universal health coverage (UHC), sustainable development goal (SDG)

## Abstract

The majority of the world's population is still facing difficulties in getting access to primary healthcare facilities. Universal health coverage (UHC) proposes access to high-quality, affordable primary healthcare for all. The 17 UN sustainable development goals (SDGs) are expected to be executed and achieved by all the 193 countries through national sustainable development strategies and multi-stakeholder partnerships. This article addresses SDG 3.8—access to good quality and affordable healthcare and two subindicators related to societal impact (SDG 3.8.1 and 3.8.2) through two objectives. The first objective is to determine whether health expenditure indicators (HEIs) drive UHC, and the second objective is to analyze the importance of key determinants and their interactions with UHC in three economic blocks: emerging Gulf Cooperation Council (GCC); developing Brazil, Russia, India, China, and South Africa (BRICS) *vis-à-vis* the developed Australia, UK, and USA (AUKUS). We use the WHO Global Health Indicator database and UHC periodical surveys to evaluate the hypotheses. We apply state-of-the-art machine learning (ML) models and ordinary least square (traditional—OLS regression) methods to see the superiority of artificial intelligence (AI) over traditional ones. The ML Random Forest Tree method is found to be superior to the OLS model in terms of lower root mean square error (RMSE). The ML results indicate that domestic private health expenditure (PVT-D), out-of-pocket expenditure (OOPS) per Capita in US dollars, and voluntary health insurance (VHI) as a percentage of current health expenditure (CHE) are the key factors influencing UHC across the three economic blocks. Our findings have implications for drafting health and finance sector public policies, such as providing affordable social health insurance to the weaker sections of the population, making insurance premiums less expensive and affordable for the masses, and designing healthcare financing policies that are beneficial to the masses. UHC is an important determinant of health for all and requires an in-depth analysis of related factors. Policymakers are often faced with the challenge of prioritizing the economic needs of sectors such as education and food safety, making it difficult for healthcare to receive its due share. In this context, this article attempts to identify the key components that may influence the attainment of UHC and enable policy changes to address them more effectively and efficiently.

## Background

The main goal of universal healthcare is to make healthcare affordable and accessible to every person on the planet without financial hardship. Universal health coverage (UHC) is a key aspect of achieving the two primary goals of the World Bank Group (WBG). The primary goal is to eradicate extreme poverty and facilitate shared prosperity. UHC is a key driver of all WBG projects related to health and nutrition. UHC focuses primarily on empowering and investing in human capital, which is the primary strength of any country. Without quality human capital, children will be unable to go to school and adults will not be able to go to work. Healthcare constitutes the largest sector of the global economy. The healthcare sector currently employs more than 50 million professionals (the majority of them are dominated by women).

Sustainable development goal (SDG) 3.8 is to facilitate UHC, which includes access to affordable healthcare services, financial risk protection, and access to safe, effective, quality medicines and vaccines for all. SDG 3.8 complements the objectives of SDG 1, whose primary goal is the eradication of poverty, which is impossible without stringent enforcement of UHC (United Nations Sustainable Development Indicators., 2019).

Immediately after the UN high-level meeting on UHC (in September 2019), it was noted that UHC is gaining global traction. Member states overwhelmingly endorsed a political declaration reaffirming their high-level political commitment to UHC and specifying the number of critical activities. In total, 12 cosignatories, including the WBG, have established the global action plan (GAP) for healthy lives and well-being for all to assist nations in collectively achieving the SDG3 objectives. Subsequently, in January 2020, the second UHC conclave was held to further boost the political involvement of UHC in the international platform.

Universal health coverage stresses access to primary health services. The most beneficial sector of UHC is human capital. Human capital, such as women, children, adolescents, and people with mental disorders, will benefit significantly. On the other hand, it is alarming to note that, if current healthcare trends continue, 5 billion people will be unable to access even primary healthcare services by 2030. Furthermore, it is also predicted that maternal and infant mortality rates will ramp up in several parts of the world. To counter this healthcare threat, the Global Financing Facility (GFF) was set up by the WBG in 2015. The main focus of the GFF is to help countries improve healthcare facilities for children, mothers, and adolescents.

The employment of the younger workforce will significantly fuel economic growth and, thereby helps in the eradication of poverty. It is the elected government's responsibility to invest in enhancing human capital. This can only be done by improving the quality of healthcare and prosperity. Enhancing reproductive, maternal, neonatal, child, and adolescent health (RMNCAH) and managing mental health issues are critical steps toward UHC. Significant problems exist, including the following:

- *Maternal Mortality:* The share of maternal mortality in developing nations is increasing year by year. It has been observed that the risk of maternal mortality is on average 1 in 56 as opposed to 1 in 7,800 in developed countries. In South Asia, 20% of maternal deaths are normally reported. Most of these deaths could be avoided if these women were given the necessary healthcare in time.
- *Child Mortality:* According to the study carried out by the World Health Organization (WHO), the World Bank, and UNICEF, there is a considerable decline in the percentage of child mortality between 1990 and 2018. It is sad to note that current healthcare data report that an average of 15,000 children under the age of 5 die every day. In addition, the WBG, WHO, and UNICEF partnered on another 2020 publication that focused on stillbirths, a problem that is often ignored. Every year, 2 million infants are stillborn worldwide, and success in lowering these numbers has lagged behind the drop in under-five mortality. In 2000, the ratio of stillbirths to deaths among children under the age of 5 was 0.30; by 2019, it had risen to 0.38 globally.
- *High Fertility*: Women are having fewer children now than they did three decades ago. Countries with high fertility frequently have high maternal, newborn, and infant mortality rates.
- *Adolescent Fertility:* In nations with high fertility rates, more adolescents give birth. Underage mothers are more likely to suffer from pregnancy problems such as obstructed labor and eclampsia, increasing their chance of mortality. Adolescent-born children are also more likely to have low birth weight, illness, stunting, and other nutritional problems.

*Mental, Neurological, and Substance Use Disorders (MNS):* These prevalent, severely debilitating illnesses are linked to considerable early mortality and impose a human, societal, and economic toll. Every 40 s, someone in the world commits suicide.

To ultimately accomplish the purpose of UHC and increase human capital results globally, mental health initiatives must be linked to community service delivery and protected by financial protection structures. According to estimates, almost 1 billion individuals have a mental condition. More than 75% of patients with the illness do not obtain treatment in low-income nations. By the age of 14, over half of all mental health illnesses have emerged, and around 20% of children and adolescents globally have some form of mental disease. More than one in every five persons (22.1%) have mental illness in nations afflicted by war and violence. Women and children who have witnessed violence, soldiers returning from the battle, migrants and refugees displaced by conflict, the destitute, and other vulnerable groups are disproportionately impacted. The COVID-19 epidemic has resulted in a global spike in mental health issues due to several variables such as anxiety, lockdowns, job losses, disturbing, or perhaps suspending, key mental health services in 93% of countries worldwide.

Mental, neurological, and substance use disorders have an early age of onset—often in infancy or early adolescence—and are disproportionately frequent in the working-age population, contributing to worldwide economic production losses estimated at $2.5–8.5 trillion, which are expected to almost three times by 2030.

The Japanese Presidency held the first-ever combined session of G20 Finance and Health Ministers in June 2019. The debate was intended to rally G20 countries around the unifying goal of funding UHC in underdeveloped countries. A World Bank analysis revealed that individuals in underdeveloped nations pay half a trillion dollars per year—more than $80 per person—out of their own pockets to receive healthcare. Such costs disproportionately affect the poor and jeopardize decades of healthcare improvement.

According to the 2019 World Bank/WHO study, nations must raise expenditure on primary healthcare by at least 1% of their gross domestic product (GDP) if the world addresses glaring coverage gaps and reaches the health objectives agreed upon under the SDGs. A lack of universal access to high-quality affordable healthcare jeopardizes countries' long-term economic prospects and renders them more vulnerable to pandemic threats.

Without immediate action, developing countries confronted with aging populations and increasing burdens of noncommunicable diseases will face increasing challenges in closing the gap between the demand for health spending and available public resources, extending patients' and their families' reliance on out-of-pocket spending^1^.

### Uniqueness

A more significant amount of analytical work is required at the global and regional block level to better understand who suffers from financial hardship, what are the causes of financial hardship, what are the consequences of financial hardship in the short- and long-term, and how households attempt to mitigate financial hardship in the short-term by borrowing or depleting their assets, Furthermore, how health system features can reduce or increase financial hardship. There is very sparse empirical work done on these problems globally and in developing countries in economic blocks like Gulf Cooperation Council (GCC) and Brazil, Russia, India, China, and South Africa (BRICS) compared to developed economies in blocks like Australia, the UK, and the USA (AUKUS). Hence, this research adds to the academic and practice literature to help policymakers take appropriate action to achieve the targets set under 3.8.1 and 3.8.2 in these economies by 2030.

### Innovation

This article applies an artificial intelligence (AI) tool through Random Forest modeling to analyze the data and demonstrates its superiority over conventional ordinary least square (OLS) regression methods traditionally found in earlier studies.

### Significance

The significance of this research includes the following contributions:

- Improving border epidemic and pandemic public health surveillance (PHS) capacities in India–UAE. This objective is reached by analyzing and contrasting the health security status of the UAE and India methodically.
- Increasing PHS workforce in India and the UAE. We are developing a capacity-building ontology framework and training curriculum (with stakeholder feedback and field testing) to manage SDG and Global Health Security Agenda (GHSA) gaps in human, animal, and environmental departments.
- Expanding India's and the UAEs' present field epidemiology capacity to better identify and mitigate diseases and pandemics in the region.
- Increasing emergency preparedness in India–UAE to better control and prevent disease outbreaks in the region.
- Improving electronic disease surveillance across India and the UAE by supporting the GHSA and deploying surveillance activities.

## Research Problem

In 2015, all the 193 UN member states accepted the 2030 Agenda for sustainable development (SD) and the 17 SDGs. This global agenda's implementation and achievement will depend on all nations and necessitate national sustainable development strategies and multi-stakeholder partnerships. Six fundamental transformations must occur to fulfill the SDGs. Each of the six transitions necessitates a significant increase in public investments. The financial requirements of these SDG initiatives exceed the budgetary space available to governments in low-income developing nations (LIDCs). To attain the SDGs, LIDCs will need to enlarge their budgetary space significantly, which would require a mix of local and global fiscal measures. This article focuses on the second transformation—access to high-quality affordable healthcare—and two sub-indicators, 3.8.1 and 3.8.2, to assure societal effect. The contribution of this manuscript would be improved health and safety outcomes through universal healthcare and would pave the path for public-sector policy change or influence.

Indicators of financial protection show inconsistent results between 2000 and 2019 in preventing people from encountering financial hardship (while paying for healthcare out-of-pocket). The number of people and the percentage of the population impoverished by out-of-pocket health spending at the thresholds of $1.90 and $3.20 per person per day. At the same time, an increasing share of the population is experiencing out-of-pocket healthcare costs. Previous worldwide studies have shown that as nations get wealthier, citizens endure greater financial difficulty due to increased reliance on out-of-pocket spending.

The policy challenge is to guarantee that increased healthcare resources are channeled through mandatory pooled prepayment arrangements rather than out-of-pocket payments, which results in access inequalities across various income strata.

### Specific Research Objectives

In the light of the foretasted problem, the specific objectives of this article are as follows:

1. To explore whether health expenditure indicators (HEI) drive the UHC index.
2. To assess the level of importance and degree of impact of HEI on UHC in emerging GCC and BRICS compared to the developed AUKUS economic block.

### Hypotheses

The research objectives are evaluated through the following sets of general and specific hypotheses.

#### General

- The higher the current health expenditure (CHE) as % of GDP for various healthcare, the higher will be the UHC.
- The higher the out-of-pocket expenses by the household for healthcare, the lower will be the UHC.
- The higher the compulsory health financing arrangements as % of CHE, the higher will be the UHC.
- The higher the government health financing arrangement as % of CHE, the higher will be the UHC.
- The higher the voluntary health financing arrangement as % of CHE, the higher will be the UHC.
- The machine learning (ML) technique is more appropriate for predicting UHC than the traditional OLS technique.

#### Specific Hypotheses

Universal health coverage is a complex function of health and non-health determinants. The influence of a given variable varies between different geographies and economies. The interactions of the variables within the model will provide a clear indication for policymakers to emphasize the same while drawing up programs for addressing the same.

Therefore, the specific hypothesis states that the importance of each input and control factor varies across the three blocks and that the impact factors (synaptic weights in the ML model) also differ across the blocks.

### Rationale for Focusing on the BRICS, GCC, and AUKUS

Brazil, Russia, India, China, and South Africa are an economic block of the world's most significant growing economies, accounting for 41% of the worldwide population, 24% of worldwide GDP, and more than 16% of universal trade (World Health Organization, 2021). The varying proportions of infectious and chronic lifestyle diseases exhibit a distinct pattern regarding morbidity and mortality across the economic blocks. The BRICS have fueled economic advancement throughout the years. BRICS nations differ substantially in terms of illness loads, healthcare systems, interests in global pharmaceutical trade, international participation, and many other factors. The rise of the BRICS as a unique organization with rising degrees of transnational cooperation in health and other activities puts pressure on existing and new global governance systems and procedures. Many of those who advocate for UHC, whether as scholars, lawmakers, or consultants, look to national governments and regional or other blocks for leadership and inspiration. Some countries that have historically supplied such leadership have mostly retreated, leaving a need that the BRICS may potentially fill. More study is needed to determine whether this gap exists, whether it is significant, and whether the BRICS can fill it (McKee et al., 2014; Watt et al., 2014; Wagstaff et al., 2018b).

To move closer to UHC, the BRICS have implemented health system changes. Despite the fact that national governments have played a significant part in these changes, private financing accounts for a significant portion of BRICS health spending. China and India rely heavily on direct expenditures, whereas Brazil and South Africa rely heavily on private insurance. Brazilian health reforms resulted from a political campaign that established health as a constitutional right. On the other hand, those in China, India, the Russian Federation, and South Africa were an attempt to enhance public sector performance and eliminate disparities in access. The transition to universal healthcare has been sluggish. Reforms in China and India have not effectively addressed the issue of out-of-pocket expenses. Negotiations between national and subnational institutions have sometimes proven difficult, but Brazil has established strong cooperation between federal and state authorities *via* a constitutional definition of authority. Poor coordination has resulted in fragmented pooling and inefficient resource usage in the Russian Federation. It is critical to utilize public and private sector resources in mixed health systems (Rao et al., 2014). The BRICS are dedicated to disseminating lessons learned from their recent experiences. These nations are progressively fostering the development of different global health programs, including UHC, by providing diplomatic support and serving as technical resources. Representatives of the BRICS nations, for example, “emphasized the significance of UHC as a key tool for the realization of the right to health” during the 65th World Health Assembly in 2012 (O'Neill, 2001).

#### Gulf Cooperation Council

The GCC has a UHC service coverage index of 72.5% (World Bank, 2021). The GCC and BRICS economies are active, vigorous, and favorable to economic divergence. As a result, they draw capital influxes from foreign investors as the GCC and BRICS countries continue to make inroads into global economics and experience faster economic development than industrialized nations mired in a slow-growth atmosphere (Bhuyan et al., 2016). According to World Federation of Exchanges statistics at the end of 2015, the total market capitalization of the GCC and BRICS nations is US$12,809 trillion, which is US$1,200 trillion more than the entire joint market capitalization of Europe, the Middle East, and Africa. Furthermore, the GCC and BRICS economies are the origins of significant sources of demand and supply.

The UAE has about 11.5% of its population as citizens, with the rest, 88.5%, made up of expatriate employees as of 2018. South Asians make up 59.4% of non-UAE nationality. Similar to developing markets, the GCC and BRICS markets are subject to macroeconomic and global market circumstances (Mensi et al., 2014).

Internal variables play an essential role in driving economic and financial conditions in the GCC and BRICS nations. There is much evidence that foreign influences drive many of the GCC and BRICS countries' economic and financial conditions. Undoubtedly, the healthy economic circumstances in the AUKUS and the rest of the developed countries benefit the economies of the GCC and BRICS, which share critical strategic commodities such as gold and crude oil. China and India are two of the world's most significant users of crude oil. On the other hand, Russia is one of the world's top crude oil and natural gas producers, with economic linkages to industrialized economies. In contrast, deteriorating economic circumstances in developed economies would decrease GCC and BRICS exports to developed markets and decrease funds and capital influxes from advanced to GCC and BRICS economies.

The GCC block is oil-rich and has enough potential to drive and lead UHC. The model adopted by the rich GCC block can be tuned and adopted by the rest of the economic blocks, especially in the BRICS countries. The BRICS is a potential unit of leading information technology collaboration. India's 2022 information technology-related exports are expected to touch new heights. A positive growth rate of 4.7% is expected to be achieved in the Indian export industry. The following are the unique predictions from the perspective of India alone:

- It is estimated to cross the US$ 400 billion mark concerning outbound shipments.
- The world trade organization (WTO) predicts that India can increase its exports by 4.7% in 2022.
- It has been estimated that software exports will cross the line of US$ 148 billion. This is more than the oil sales of the GCC countries.
- In the coming months of 2022, India is expected to experience exponential growth in software companies.

Because the BRICS have the world's most significant engineering population, the software export business in the BRICS has the potential to soar in 2022 and thereafter. Although software exports are a component of export-led growth stories, they appear to be gaining traction in recent years.

Australia, the UK, and the USA block is a trilateral technology accelerator between the governments of the three signatory nations by accelerating the development and application of critical technologies in the hands of their servicemen and women. It is a trilateral agreement that is bringing three other joined “trilateral” in each of the three nations: between governments, research organizations, and companies—including tech firms outside the traditional defense sector. AUKUS is deeply complementary to the QUAD (an alliance of four countries, Australia, the USA, Japan, and India, formed in 2007), and a foundational contribution to a free, open, and inclusive Indo-Pacific. While AUKUS focuses on security issues, QUAD discusses diplomatic and global issues, including the COVID-19 situation, vaccines, technological innovation, supply chain resilience, and climate change.

## Literature Review

Several nations have implemented UHC-inspired health reforms, and UHC has been identified as one of the new SDGs (Horton and Das, 2015). Moreover, regardless of their financial ability to pay, everyone obtains the healthcare they require without undue financial hardship (Boerma et al., 2014). Investing in more comprehensive UHC and primary care can improve population health and reduce health disparities. Trade-offs do occur between health policy objectives. Healthcare technologies, policies, and resources should be subjected to distributional analysis (Cookson et al., 2021). Particularly, with the United Nations SDGs 2030, Goal 3, “Indicator 3.8.1”: Coverage of essential health services and “Indicator 3.8.2”: Proportion of population with large household expenditures on health as a share of total household expenditure or income are vital areas of focus (UN-SDG, 2019). Measuring progress toward UHC, thus, entails keeping track of both the service coverage and financial security components of UHC. An earlier study (Hogan et al., 2018) on summarizing national levels of service coverage to track progress toward UHC either concentrated on specific regions or relied on mortality-based metrics that are imprecisely recorded in the majority of developing nations. The monitoring framework for SDGs calls for the creation of an index of critical service coverage to track progress toward SDG goal 3.8 on universal healthcare across nations (Hogan et al., 2018).

However, except for two studies (Wagstaff et al., 2015, 2016), works have evaluated each dimension of UHC separately. Such studies, as noted by Hogan et al. (2018), Wagstaff et al. (2018a), and possibly misleading (McPake BI., 2018), are potentially misleading as nations may perform well on one UHC component but not the other. Low out-of-pocket expenditure (OOPS) on health might indicate that individuals are not getting the treatments they require or they are receiving these services but are not paying for them out-of-pocket. Furthermore, a high level of use of health services may or may not be related to a high degree of out-of-pocket cost.

“Machine learning is the most visible expression of AI and the newest development area in digital technology, promises to accomplish more with less and might be the catalyst for such a shift (PubMed Central (PMC), 2018). However, the nature and scope of this commitment has [sic] not been thoroughly evaluated”. “To overcome these problems and attain universal health coverage (UHC) by 2030, a fundamental restructuring of health systems is necessary”.

On many occasions, health and healthcare are often linked, the former as a direct function of the latter. However, attaining the level of healthcare services that guarantee optimal health is a mirage for most countries. Though health is a state subject that requires the government to ensure the delivery of healthcare to the population, the reality of UHC remains an unrealized dream. The situation in the Indian context is complicated by the enormous population and the prevalence of infectious and noncommunicable diseases burden. Additionally, the issues of gender equity, the predominance of the private health sector for secondary and tertiary care, maldistribution of trained human resources, and economic factors complicate attaining desired levels of UHC. Policy requirements at the national and state level need to be guided by research to identify the core determinants that have a bearing on UHC (Singh, 2013).

Health indicators, such as maternal mortality ratio, infant mortality rate, and under-five mortality rates, are sensitive to factors related to healthcare delivery systems. However, morbidity indicators reflect UHC more realistically as mortality is not an outcome of several diseases, especially chronic illness. Across the globe, governments are trying to ensure optimal access and affordable healthcare for populations. The costs of healthcare are rising in the wake of modern and diagnostic advances in healthcare. Though primary healthcare is essential to ensure the health and wellbeing of the masses, the availability of secondary and tertiary curative and rehabilitative networks completes the circle of healthcare. With the emergence of mental health issues as an additional burden of morbidity that requires early diagnosis and appropriate management, healthcare systems are getting overwhelmed. In this context, policymakers need to identify modifiable determinants to ensure universal healthcare and promulgate affirmative actions toward achieving the same. The sustenance of interventions needs to be considered for long-term benefits to the system. A multi-sectoral approach with the cooperation and involvement of non-health players is essential to achieve universal healthcare for the population (Kumar, 2020).

### Proposed Framework

**Figure 1:**
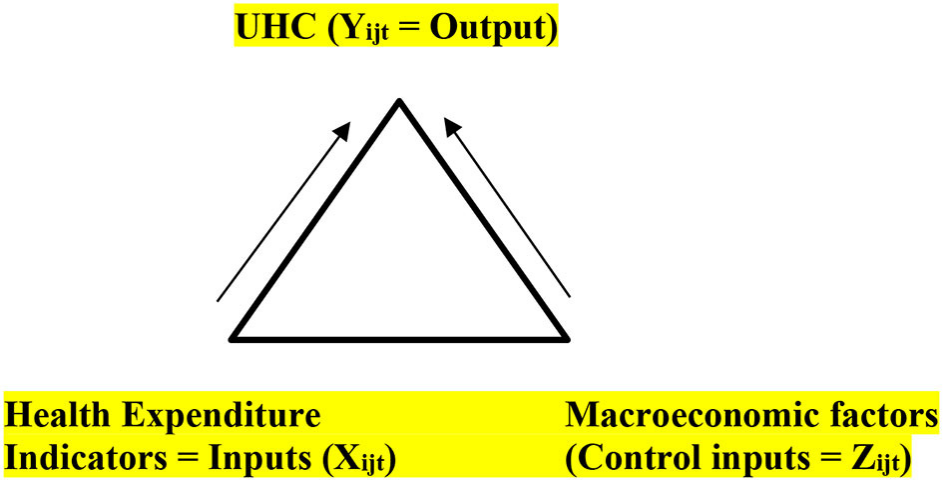
Conceptual framework. Input-output relation of universal health coverage (UHC) with health expenditure and macroeconomic factors.

The suggested conceptual framework is an output-input framework, as depicted in Figure 1. As stated in earlier sections, UN SDG Target 3.8-achieving UHC is a primary objective, which includes financial risk protection, easy accessibility to most essential health services, access to adequate quality, affordable, most essential medications, and vaccinations for everyone. It is essential to reach everyone who requires health-related services (including health services, medicines, and other resources). Healthcare services must be served without spending out-of-pocket and without witnessing financial hardships. To achieve this, two indicators are identified under 3.8—namely, 3.8.1 and 3.8.2. The 3.8.1 indicator focuses on UHC, and the 3.8.2 indicator focuses significantly on healthcare-related financial hardship/burden on patients. The two indicators in 3.8 primarily represent healthcare service coverage and financial protection. Reproductive facilities, newborn care, child healthcare, handling of medication for infectious diseases, and treatment of communicable/noncommunicable diseases are covered by essential health coverage. These are the categories that represent the most disadvantaged populations. The indicators used to measure the effectiveness of implemented policies are based on a scale of 0–100. This unit of measurement has no unit. These indicators are derived from 14 various healthcare coverage factors. Thus, in the framework, the purpose is to predict UHC (the output *Y*_*it*_) in different blocks, and how they are influenced by various factors (the inputs *X*_*it*_ and the control factors *Z*_*it*_).

Figure 1 succinctly encompasses the input-output analysis to address the research objectives. The input and control input factors are detailed in Table 1.

**Table 1:** Details of Output (Y_ijt_) and Inputs (X_ijt_ and Z_ijt_).

**Y/X_**i**_**	**Variable name**
Y	Universal Health Coverage
X_1_	Current Health Expenditure (CHE) as % Gross Domestic Product (GDP)
X_2_	Current Health Expenditure (CHE) per Capita in US$
X_3_	Voluntary Health Insurance (VHI) as % of Current Health Expenditure (CHE)
X_4_	Out-of-pocket (OOPS) as % of Current Health Expenditure (CHE)
X_5_	Out-of-Pocket Expenditure (OOPS) per Capita in US$
X_6_	Compulsory Financing Arrangements (CFA) as % of Current Health Expenditure (CHE)
X_7_	Government Financing Arrangements (GFA) as % of Current Health Expenditure (CHE)
X_9_	Voluntary Financing Arrangements (VFA) as % of Current Health Expenditure (CHE)
X_17_	Current Health Expenditure (CHE)
X_18_	Domestic General Government Health Expenditure (GGHE-D)
X_19_	Domestic Private Health Expenditure (PVT-D)
**Macro factors**	
Z_1_	GDP-constant 2019 US$ reflecting the magnitude of the country's economy in year t
Z_2_	Inflation reflecting purchase power parity of the country in year t
Z_3_	Population reflecting size of the country in year t

The framework is empirically analyzed using the following traditional OLS and ML specifications:

Empirical model (traditional OLS):


(1) $$\begin{array}{r} Y_{jjt}=\alpha_{ijt}+\beta_{ijt}\sum \sum \sum {X_{ijt}}_{+}\pi_{ijt}\sum \sum \sum Z_{ijt}+{\text{Error}}_{ijt} \end{array}$$

where *Y*_*ijt*_ = output = UHC index service coverage for countries in the three blocks *j* and *t* = 2017, 2019.

*X*_*ijt*_ = Set of healthcare indicators inputs for countries in the three blocks *j* and *t* = 2017, 2019.

*Z*_*ijt*_ = Set of macro factors such as GDP (2019 constant US$), inflation, and size of the country represented by population for countries in the three blocks *j* and *t* = 2017, 2019.

*J* = 14 economies grouped into three economic blocks (represented by indicators 1, 2, 3), where 1 represents the BRICS block (five developing economies): Brazil, Russia, India, China, and South Africa); 2 represents the GCC block (six developing oil-rich economies: Bahrain, Kuwait, Oman, Qatar, Saudi Arabia, and the UAE); 3 represents the AUKUS block (three developed economies: Australia, the UK, and the USA); and *t* = time indicator (2017, 2019) years where full and consistent data are available for all the 14 countries.

### Data

The indicators above are collected from the WHO Global Health Indicator database and UHC periodical surveys of 2017 and 2019 for the 14 countries stated earlier (survey data were not complete for all 14 countries for 2005 and 2016 and, hence, were not wholly included in the analysis). The following hypotheses are tested in both OLS and ML specifications.

### Data Limitations

Because of data constraints, not all tracer indicators used to calculate the UHC index are direct service coverage measurements. The chosen tracer indicators indicate the broad range of primary healthcare required for progress toward UHC; they should not be taken as a suggested basket of services. The WHO data were curated with the following attributes.

1. Since UHC data are available for 2017 and 2019, we have focused on these 2 years' indicators and macro factors. Therefore, all years before 2017 were excluded. Wherever the information for 2017 or 2019 was missing, the data were extrapolated by taking the average of the survey data from the prior 5 years.
2. Furthermore, there were no granular data in specific healthcare categories in developed countries. Therefore, these components were excluded from the data set.

### ML Methodology

Since we have only 2 years of UHC and *X*_*i*_ data for all block countries, applying traditional OLS will be less valuable as the degree of freedom will be very low^2^. Hence, we use ML algorithms similar to regression in concept, viz., “Random Forest regressor,” a Decision Tree method.

### Overview of Random Forest Tree Method

Random Forest is an ensemble learning technique for classification, regression, and other problems that are trained with many decision trees. Because the decision tree is a simple method, a single tree may not be sufficient for the ML model to acquire its features. On the other hand, Random Forest is a “Tree”-based algorithm that produces decisions by blending the characteristics of many decision trees. As a result, it is possible to characterize it as a “Forest” of trees, hence the term “Random Forest.” This approach is a forest of “Randomly Created Decision Trees,” as the name implies.

Overfitting is a crucial disadvantage of the decision tree approach. However, this difficulty may be minimized by employing the Random Forest regression instead of the decision tree regression. Furthermore, the Random Forest approach is more robust and speedier than traditional regression models. To summarize, the Random Forest algorithm combines the outcomes of many decision trees to get the final result.

A Random Forest is an ensemble approach in AI that performs prediction and classification by collating several trees using bootstrap (sometimes referred to as bagging). Instead of considering one decision tree, the main idea is to aggregate numerous trees to decide the outcome.

We randomly choose rows and features from the data set to create sample data sets for each model. This section is known as bootstrap. We must approach the Random Forest regression technique in the same way as we would any other ML technique:

- Create a specific query or data set and work with the source to determine the relevant data.
- Ensure that the data are in an easily accessible format. Otherwise, convert them to the appropriate forum.
- Specify any apparent abnormalities or missing data points necessary to obtain the relevant data.
- Develop an ML model.
- Determine the baseline model you wish to accomplish.
- Train the ML model using the data.
- Using the test data, provide insights into the model.
- Now compare the performance metrics of the test data with the model's projected data.
- If it does not meet researchers' expectations, we may improve the model by dating our data or using another data modeling approach.
- Interpret the data at this point.
- At this point, analyze the data gathered by researchers and report accordingly.

## Results

### Radom Forest Tree Results

Table 2 shows the features that influence UHC after applying the abovementioned technique.

**Table 2:** Basic Random Forest-Based feature importance for UHC coverage covering all the three blocks.

**Feature name**	**Variables name**	**Random forest feature (RFF)**	**RFF importance**
X5	Out-of-Pocket Expenditure (OOPS) per Capita in US$	0.23952136	1
X19	Domestic Private Health Expenditure (PVT-D)	0.189957	2
X18	Domestic General Government Health Expenditure (GGHE-D)	0.1155865	3
X2	Current Health Expenditure (CHE) per Capita in US$	0.09970508	4
X17	Current Health Expenditure (CHE)	0.08736607	5
Z1	GDP-constant 2019 US$	0.06472806	6
X1	Current Health Expenditure (CHE) as % Gross Domestic Product (GDP)	0.0646806	7
X6	Compulsory Financing Arrangements (CFA) as % of Current Health Expenditure (CHE)	0.03737473	8
X7	Government Financing Arrangements (GFA) as % of Current Health Expenditure (CHE)	0.03265779	9
Z3	Population	0.02063635	10
X9	Voluntary Financing Arrangements (VFA) as % of Current Health Expenditure (CHE)	0.01583275	11
X3	Voluntary Health Insurance (VHI) as % of Current Health Expenditure (CHE)	0.01494308	12
X4	Out-of-pocket (OOPS) as % of Current Health Expenditure (CHE)	0.01103154	13
Z2	Inflation	0.0059791	14

**Figure 2:**
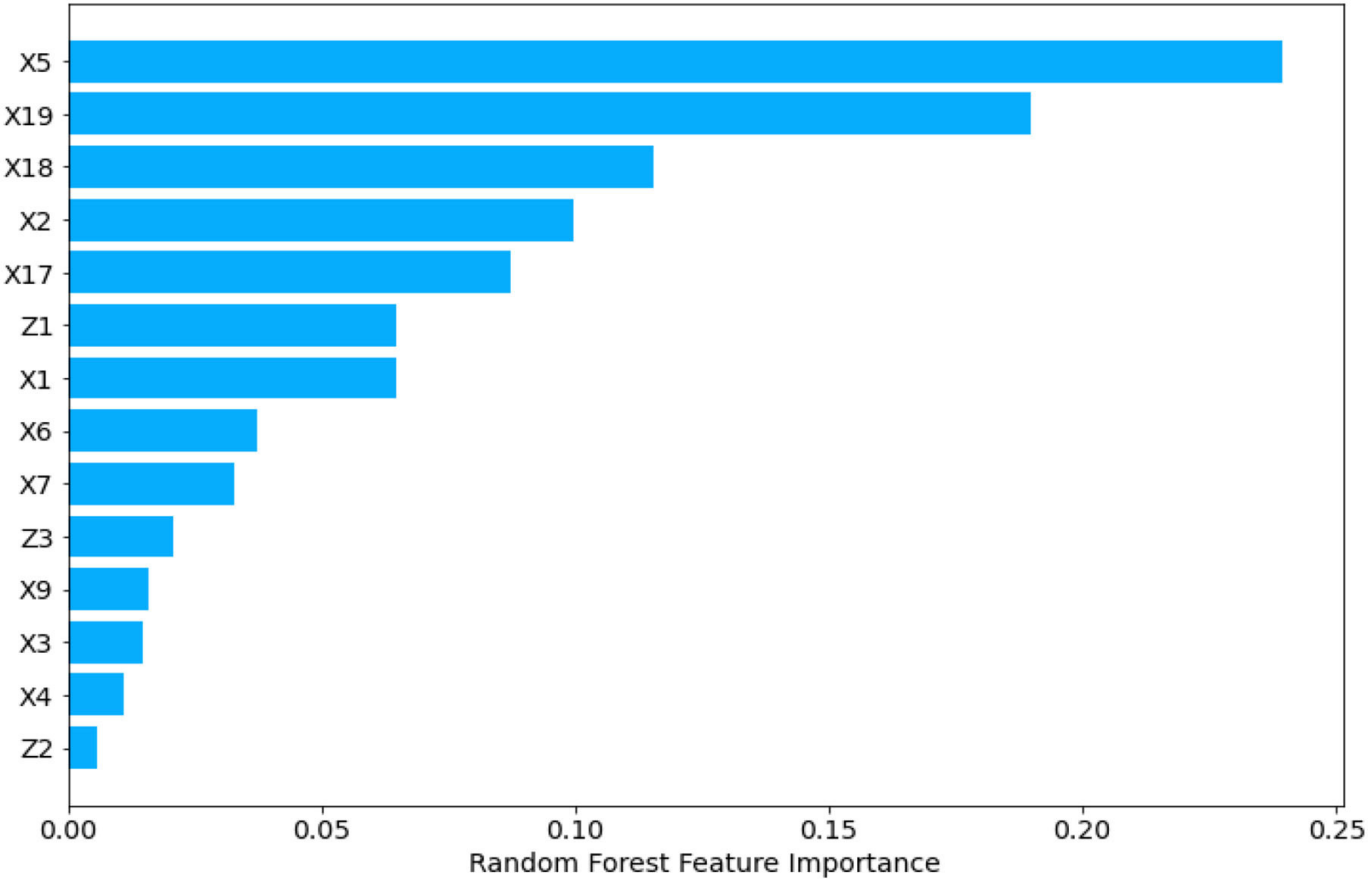
Random Forest feature importance in UHC for all three economic blocks.

For all the blocks, UHC is impacted by:

- OOPS per Capita in US$ with the importance of 23.95% *(General Hypothesis 2 is validated)*.
- PVT-D with the importance of 18.99%.
- Domestic government health expenditure (GGHE-D) with the importance of 11.56%.
- CHE per Capita in US$ with the importance of 9.97%.
- CHE with the importance of 8.74%.
- GDP-constant 2019 US$ (magnitude of the economy) with the importance of 6.47%.
- CHE as a percentage of GDP is 6.47% *(General Hypothesis 1 is validated)*.
- Compulsory Financing Arrangements (CFA) as a percentage of CHE with a weight of 3.74% *(General Hypothesis 3 is validated)*.
- Government Financing Arrangements (GFA) as a percentage of CHE with a weight of 3.27% *(General Hypothesis 4 is validated)*.
- Population (density of human capital in the economy) with the importance of 2.1%.
- Voluntary Financing Arrangements (VFA) as a percentage of CHE with a value of 1.58% *(General Hypothesis 5 is validated)*.
- Voluntary health insurance (VHI) as % of CHE with the importance of 1.49%.
- OOPS expenditure as a percentage of CHE with a significance of 1.1%.
- Inflation with the significance of 0.006%.
- This validates our specific hypotheses that the importance of each input and control factor varies across the three blocks for UHC prediction.
- The Random Forest-based results depicted in Table 2 are graphically illustrated for all the blocks, and the same is shown in Figure 2.

### Shapley Additive Explanations to Understand the Results Produced by Random Forest Techniques

SHapley Additive exPlanations (SHAP) is most likely at the cutting edge of ML explainability. Lundberg (2017) initially released this technique, which is an excellent approach to reverse engineer the output of any prediction algorithm. SHAP values are utilized when researchers have a sophisticated model (a gradient boosting, a neural network, or anything that accepts some characteristics as the input and makes predictions as the output) and understand the model's decisions.

SHapley Additive exPlanations values are derived from Shapley values, a game theory term. For example, consider the following scenario: we have a predictive model; the “game” and the “players.” SHAP quantifies the contribution of each feature to the model's prediction. It is critical to emphasize that a “game” refers to a single observation, one observation per game. SHAP is all about a prediction model's local interpretability. The SHAP value plot can further show the positive and negative relationships of the predictors (*X*_*i*_) related to the target variable (in our case, UHC). This graphic plot is constructed using all of the dots in the train data. It displays the following data:

*Importance of feature*: Variables are ordered descending.

*Impact*: The horizontal placement indicates whether the value's influence is related to a higher or lower forecast.

*Original price*: The color indicates whether the variable of that observation is high (in red) or low (in blue).

**Figure 3:**
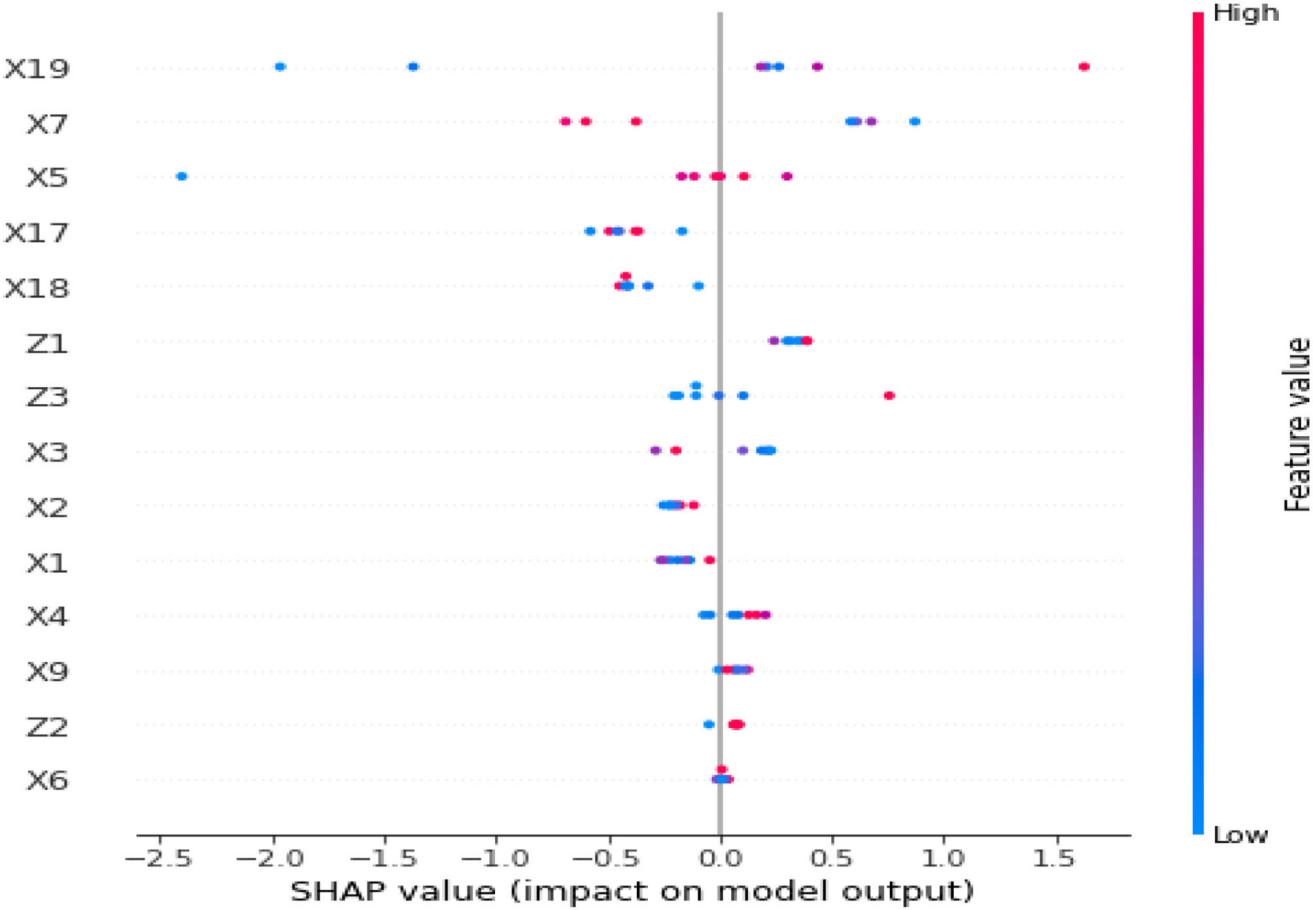
SHapley Additive exPlanations (SHAP) value for UHC prediction in all the blocks.

Figure 3 depicts the SHAP values for all blocks in our UHC forecast. The red hue represents the “high” effect.

We can quantify the contribution of the input characteristics to the individual predictions by using the SHAP values in the model explanation. The *x*-axis in this chart represents the SHAP value, while the *y*-axis contains all of the characteristics. Each point on the graph represents a single SHAP value for a prediction and feature. The color red denotes a greater value for a characteristic. The blue color denotes a lower value for a characteristic. Based on the distribution of the red and blue dots, we can obtain a basic sense of the influence of characteristics on directionality. We shall practically verify the results of the SHAP values shown in Figure 3. In this graph, based on the previous explanation, we can make the following interpretations, for example:

- The higher the value of X19 [domestic private health expenditure (PVT-D)], the higher the value of UHC.
- It can be interpreted from the feature values of X5 (OOPS) to have a negative bearing on the full realization of the optimal UHC potential for a given country.

**Figure 4:**
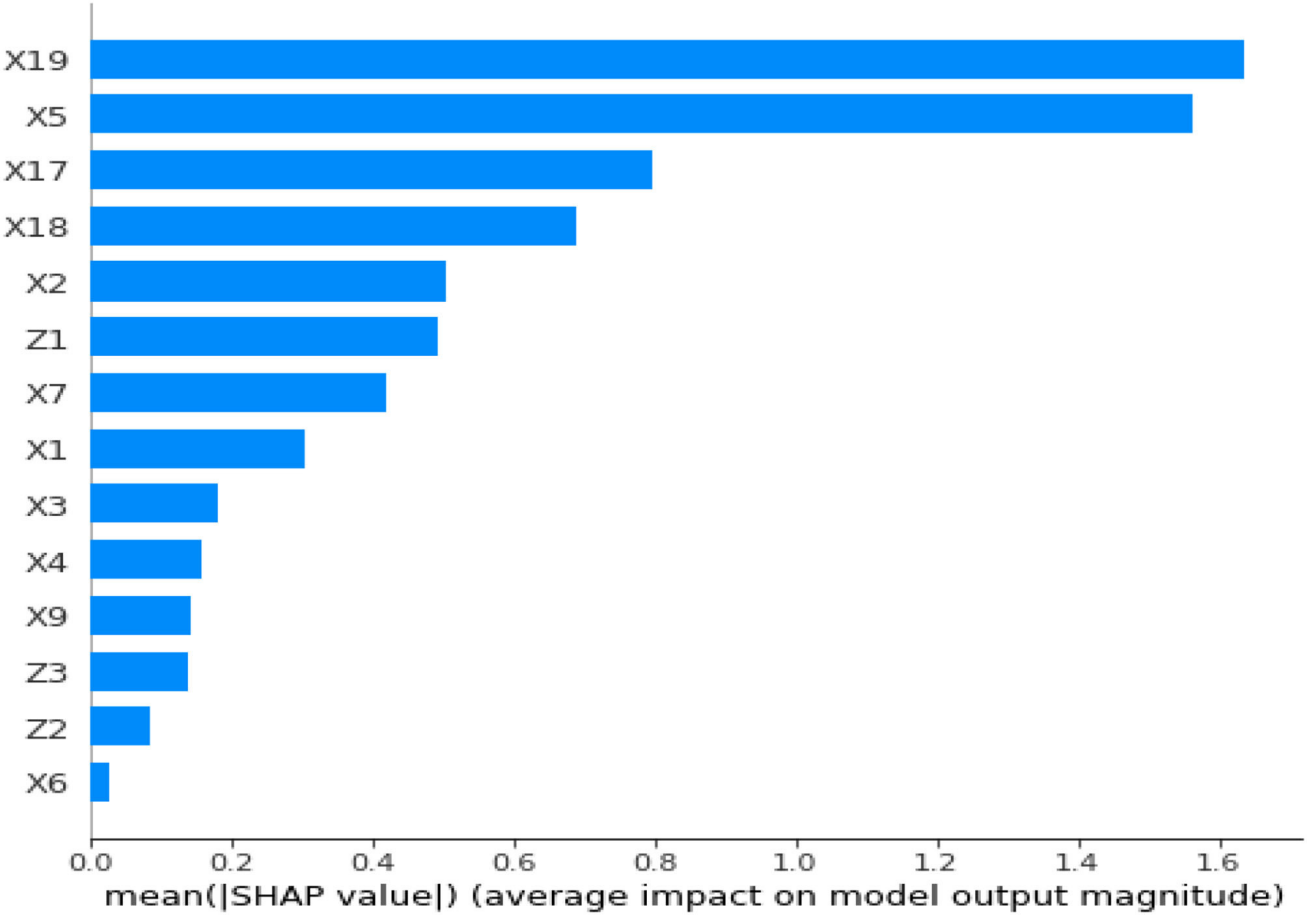
Mean SHAP values of predictors in UHC in all the blocks.

Similarly, with the help of the SHAP visualization, one can interpret the impact of feature values (either positive or negative) on the predictive variable (in our case, high or low UHC). Figure 4 shows the mean SHAP values for UHC prediction in all the blocks.

Figure 4 shows a slight shift in the percentage relevance of UHC compared to Figure 2.

### Comparing the Error Component in Terms of Root Mean Square Error

**Figure 5:**
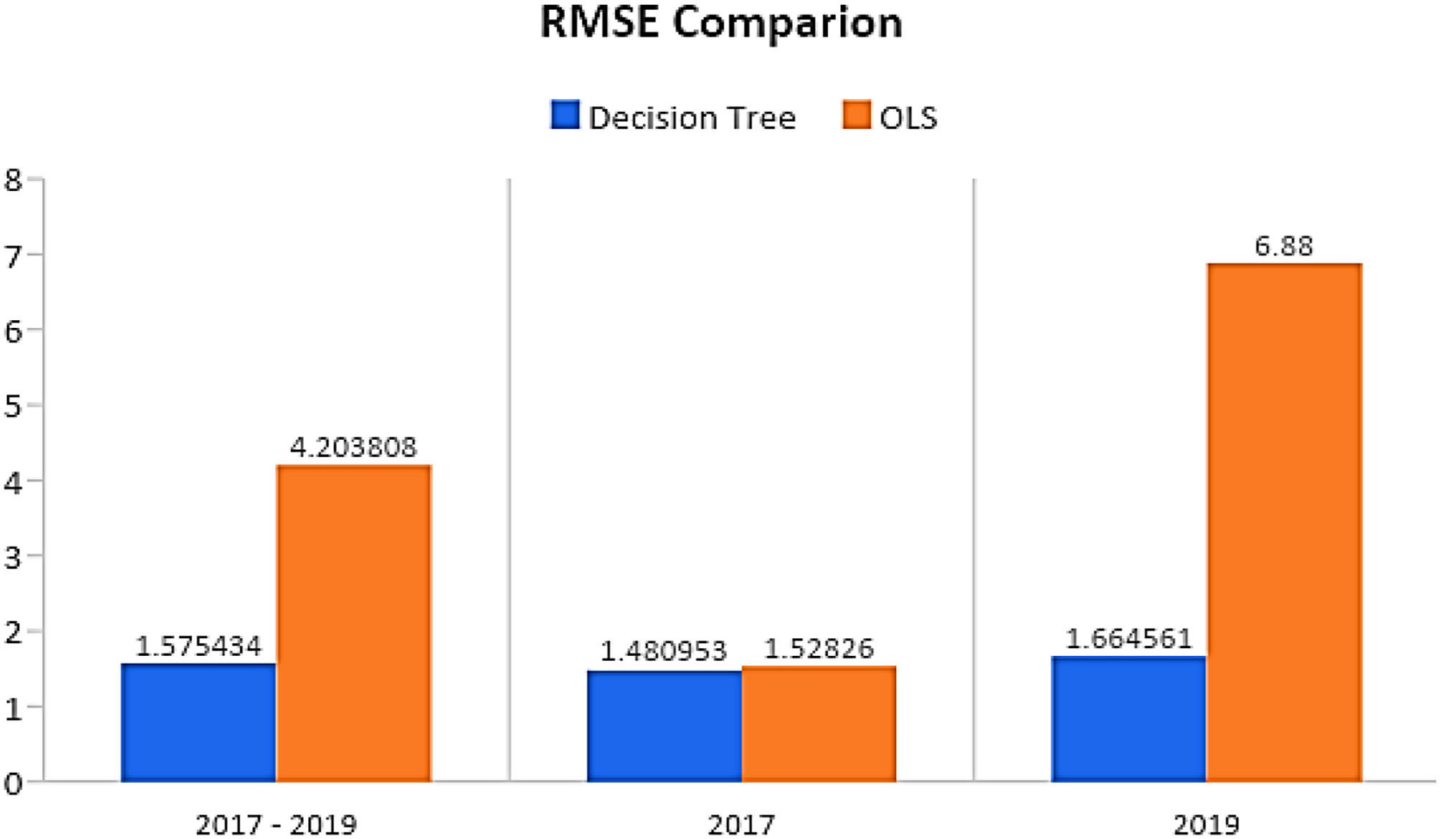
Root mean square error (RMSE) comparison between Random Forest Decision Tree ML method and ordinary least square (OLS).

The results in Figure 5 illustrate the root mean square error (RMSE) values of the methods used in this study. We have used OLS and Random Forest-based algorithms for UHC prediction. This validates our *general Hypothesis 6 that the ML technique is more appropriate for predicting UHC than the traditional OLS technique*. The result illustrated in the graph firmly conveys that Random Forest performs has superior performance to the traditional OLS model shown. Furthermore, Random Forest predictions have less residual than the OLS model.

**Figure 6:**
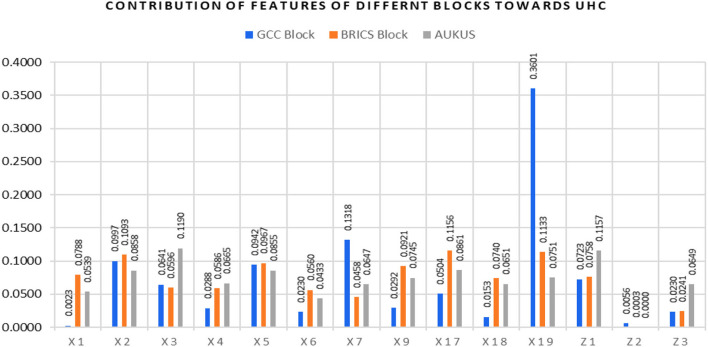
Contribution of the features for UHC in each block.

In Figure 6, the less developed BRICS economic block predictors are shown in Table 3. The predictors are arranged in decreasing order of importance for UHC. This validates our *specific hypotheses that the importance of each input and control factor varies across the three blocks, and the impact factors (synoptic weights in the ML model) also differ across the blocks for predicting UHC*.

**Table 3:** Less developed BRICS economic block predictors.

**X17**	Current Health Expenditure (CHE) Constant 2019 US$
**X19**	Domestic Private Health Expenditure (PVT-D)
**X2**	Current Health Expenditure (CHE) per Capita in US$
**X9**	Voluntary Financing Arrangements (VFA) as % of Current Health Expenditure (CHE)
**X5**	Out-of-Pocket Expenditure (OOPS) per Capita in US$
**X1**	Current Health Expenditure (CHE) as % Gross Domestic Product (GDP)
**X18**	Domestic General Government Health Expenditure (GGHE-D)
**Z1**	GDP-constant 2019 US$ (reflecting richness of the block)
**X3**	Voluntary Health Insurance (VHI) as % of Current Health Expenditure (CHE)
**X4**	Out-of-pocket (OOPS) as % of Current Health Expenditure (CHE)
**X6**	Compulsory Financing Arrangements (CFA) as % of Current Health Expenditure (CHE)
**X7**	Government Financing Arrangements (GFA) as % of Current Health Expenditure (CHE)
**Z3**	Population (Size of the economy)

*The predictors are arranged in decreasing order of importance for UHC coverage*.

Inflation was not a key feature in the BRICS block to expand UHC. The BRICS economies must expand proactively health financing through the private sector, insurance sector, and government sector from the policy perspective. There is an incentive for individual households to increase their OOPS for getting full health coverage. The economy's health coverage strategy was undertaken from the feature importance in the oil-rich GCC block discussed below. Oil-rich GCC economic blocks had the predictors, as shown in Table 4. The predictors are arranged in decreasing order of importance for UHC.

**Table 4:** Oil-rich GCC economic blocks had the predictors.

**X19**	Domestic Private Health Expenditure (PVT-D)
**X7**	Government Financing Arrangements (GFA) as % of Current Health Expenditure (CHE)
**X2**	Current Health Expenditure (CHE) per Capita in US$
**X5**	Out-of-Pocket Expenditure (OOPS) per Capita in US$
**Z1**	GDP-constant 2019 US$ (reflecting richness of the block)
**X3**	Voluntary Health Insurance (VHI) as % of Current Health Expenditure (CHE)
**X17**	Current Health Expenditure (CHE) Constant 2019 US$
**X9**	Voluntary Financing Arrangements (VFA) as % of Current Health Expenditure (CHE)
**X4**	Out-of-pocket (OOPS) as % of Current Health Expenditure (CHE)
**Z3**	Population (Size of the economy in the block)
**X6**	Compulsory Financing Arrangements (CFA) as % of Current Health Expenditure (CHE)
**X18**	Domestic General Government Health Expenditure (GGHE-D)
**Z2**	Inflation

*Predictors are arranged in decreasing order of importance for UHC coverage*.

Oil-rich economies of the GCC are way ahead of less developed BRICS economies in expanding UHC by promoting private, compulsory, voluntary, insurance, and government participation. It also makes sense as 80% of the GCC population are expats and get permission to work as a resident in these economies. The minimum requirement is to have health insurance, either from private insurers or from their contribution as a requirement for obtaining a work permit. Are these health service measures on par with the developed economies in AUKUS? Let us examine this in the next section. The developed AUKUS economic block had the predictors in decreasing order of importance for UHC in Table 5.

**Table 5:** Developed AUKUS economic block had the predictors in decreasing order of importance for UHC coverage.

**X3**	Voluntary Health Insurance (VHI) as % of Current Health Expenditure (CHE)
**Z1**	GDP-constant 2019 US$ (reflecting richness of the block)
**X5**	Out-of-Pocket Expenditure (OOPS) per Capita in US$
**X2**	Current Health Expenditure (CHE) per Capita in US$
**X17**	Current Health Expenditure (CHE) Constant 2019 US$
**X19**	Domestic Private Health Expenditure (PVT-D)
**Z3**	Population (Size of the economy in the block)
**X18**	Domestic General Government Health Expenditure (GGHE-D)
**X7**	Government Financing Arrangements (GFA) as % of Current Health Expenditure (CHE)
**X4**	Out-of-pocket (OOPS) as % of Current Health Expenditure (CHE)
**X1**	Current Health Expenditure (CHE) as % Gross Domestic Product (GDP)
**X6**	Compulsory Financing Arrangements (CFA) as % of Current Health Expenditure (CHE)

Due to their highly developed status, productive resources (both physical, financial, and technological), and best health service strategies implemented by these economies, UHC is the highest in the AUKUS block compared to the BRICS and GCC blocks. The GCC has leveraged the best practices of the AUKUS to expand UHC in their economies. The abovementioned interblock analysis concludes that the economies of the BRICS should proactively follow health coverage strategies and financing arrangements prevalent in the GCC and AUKUS blocks.

It is demonstrated that the current study on UHC with respect to the three economic blocks has identified the inter- and intra-block significant (and less significant) factors. The identified factors will act as a feedback input to fine-tune UHC-related policy modifications. The decision for improvement can be informed. The focus of this research is 2-fold: to identify the critical UHC-related inter- and intra-block features and to obtain the superiority of AI ML-oriented techniques in relation to traditional Statistical bound techniques. The implemented decision tree models have shown that the prediction of UHC can be significantly improved with AI ML methods. From the perspective of the Random Forest algorithm, we are able to derive important features for inter-/intra-block UHC calculation and residual prediction calculation. The results are sustainable up to the current time, considering the advances in the analytical domain.

## Limitations

Some limitations and concerns with the index, both at the international and national levels, must be resolved in future revisions before it can be considered complete. While defining the tracer indicators, it was discovered that there was a data scarcity for the global measurements of healthcare coverage (SDG indicators 3.8.1 and 3.8.2). However, the goal was to select measures of effective service coverage, as most of the currently available index indicators measure contact coverage rather than effective service coverage. As a result, coverage measures were not selected as tracer indicators for several essential health sectors. For example, meaningful indicators of coverage of interventions for noncommunicable illnesses, mental health, injuries, and emergencies are currently scarce in most countries. In these areas, proxy measures of service coverage were used in place of coverage indicators, such as data on service capacity or health status, to determine whether services were being provided.

## Directions for Future Research

An increased effort in data collection, particularly national health examination surveys that measure the service coverage across the different domains of health, and the amount of money spent on health by households, will significantly improve countries' ability to track progress toward UHC and to complete sub-national assessments of UHC, which are likely to be the most useful ones to national policymakers in the long run. Future research can exploit these surveys efficiently toward SDG 3.8.1 3.8.2 and UHC issues.

## Conclusion

Policymakers must identify the core modifiable determinants of UHC for framing appropriate policies to ensure optimal coverage. The steps that need to be taken to alter health spending require a complex set of strategies and the involvement of several sectors. Increasing healthcare spending as a higher proportion of GDP is essential to tackle the economic determinants of UHC. To attain the target eight of SDG three, it is imperative for the healthcare providers to identify the cost centers of healthcare and address them effectively. PVT-D, OOPS per Capita in US$, and VHI as a percentage of CHE are the key factors influencing UHC across the blocks, according to the integrated models of the three economic blocks. Federal funding of comprehensive healthcare is highly challenging due to several complex features of individual healthcare systems worldwide. Largely, policies that address the issues of enhancing healthcare financing through the contribution of individuals and with the support of the federal funding structure show promise for the future.

Additionally, efforts toward containing the private expenditure for health through enhanced preventive primary healthcare would benefit UHC across different spectrums and geographies. In addition, the cost(s) incurred for primary preventive care are less than those of tertiary curative interventions. The preventive paradox makes the costs of curing existing morbidities (secondary prevention strategies) a considerable challenge as compared to primary prevention to restrict the emergence of the diseases in the first place. In this context, it is proposed that sufficient emphasis and funding allocations are made in the policies for preventive, curative, and rehabilitative healthcare services to achieve UHC.

## Author's Note

Economic block is formed by a set of countries with common long-term and immediate goals. These countries normally engage in international trades with each other and other associations related to their growth.

Brazil, Russia, India, China, and South Africa compose the BRICS economic bloc, which is made up of emerging economic powers and political influencers. These countries account for 40 percent of the world's population and 20 percent of its gross domestic output (GDP). The BRICS group's mission is to promote peace, security, development, and collaboration. The BRICS countries argue for five universal principles: sovereignty, unity, independence, territorial integrity, non-aggression, and equality. These five nations do have certain characteristics: they are all huge, populous, and varied, with numerous ethnic, socioeconomic, and - in some cases - religious differences. They share similar traits with a number of other nations, including Indonesia, Nigeria, and Pakistan, who have made less progress toward UHC but may benefit from the BRICS' experiences. There is little question that collaboration and shared learning are essential in the promotion of UHC (McKee et al., 2014).

Bahrain, Kuwait, Oman, Qatar, Saudi Arabia, and the United Arab Emirates comprise the Gulf Cooperation Council (GCC) (UAE).

The formation of AUKUS - a “enhanced trilateral security partnership” involving Australia, the United Kingdom, and the United States that will be launched in September 2021 - has fuelled the already growing momentum toward monoliteral cooperation in the Indo-Pacific in order to “meet the challenges of the twenty-first century.” The deal has broad objectives, including encouraging more information and technology exchange, integrating security and defence-related research, technology, industrial bases, and supply chains, and improving the three countries' combined capabilities and interoperability. Despite being exclusive to the three Anglo partners, the grouping has the potential to provide enormous value to the Indo-Pacific regional security architecture (Jagannath, 2022).

## Data Availability Statement

The original contributions presented in the study are included in the article/Supplementary Material, further inquiries can be directed to the corresponding authors.

## Author Contributions

All authors listed have made a substantial, direct, and intellectual contribution to the work and approved it for publication.

## Conflict of Interest

The authors declare that the research was conducted in the absence of any commercial or financial relationships that could be construed as a potential conflict of interest.

## Publisher's Note

All claims expressed in this article are solely those of the authors and do not necessarily represent those of their affiliated organizations, or those of the publisher, the editors and the reviewers. Any product that may be evaluated in this article, or claim that may be made by its manufacturer, is not guaranteed or endorsed by the publisher.

## Footnotes

^1^ https://www.worldbank.org/en/topic/universalhealthcoverage#1

^2^This was verified by the OLS model results but not reported here for brevity.
