# Supplementary material for: Predicting Universal Healthcare Through Health Financial Management for Sustainable Development in BRICS, GCC, and AUKUS Economic Blocks
Source: Front Artif Intell. 2022 Apr 29;5:887225. doi: 10.3389/frai.2022.887225 (PMC9100561; doi:10.3389/frai.2022.887225)
Supplement: Supplementary file 1 [file Data_Sheet_1.pdf]

Predicting Universal Health Care (UHC) through Health Financial Management (HFM) for Sustainable Development in BRICS, GCC, and AUKUS Economic Blocks

Appendix

A Random Forest is a collection of decision trees. This is to state that a Random Forest is made up of several trees that are built in a "random" manner.

- Each tree is built from a distinct sample of rows, and at each node, a different sample of characteristics is chosen for splitting.
- Each tree produces a prediction, which is then averaged to get a single result.

As previously stated, averaging improves the accuracy and reduces overfitting in a Random Forest over a single Decision Tree. A Random Forest Regressor forecast is an average of the predictions made by the trees in the forest. For simplicity, an example of two trees from our UHC prediction is provided below.

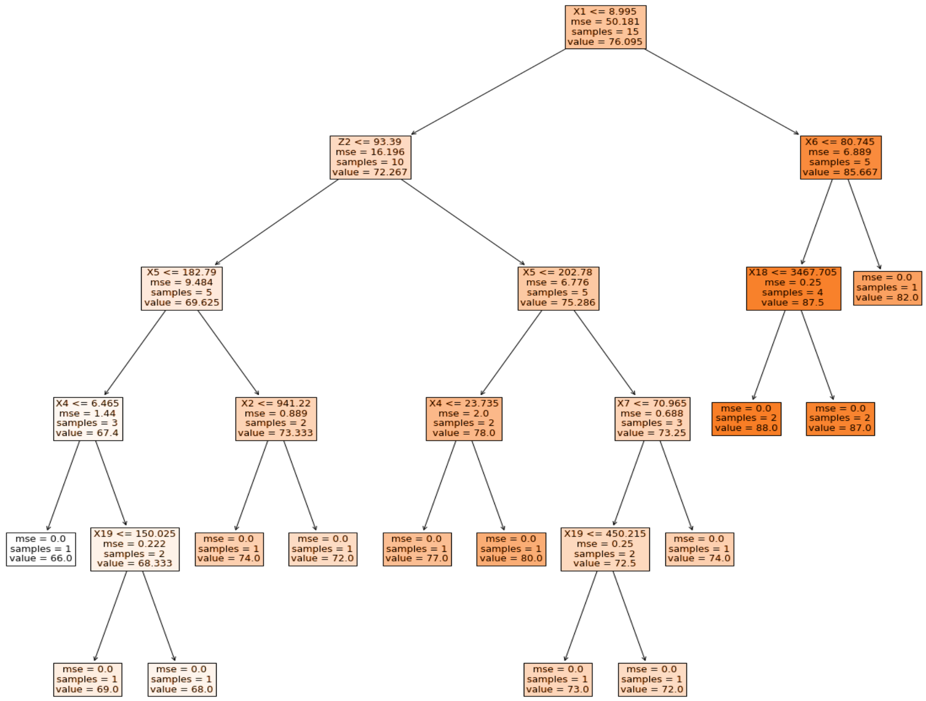

Figure A1. Example of one of the 100 discission trees produced for calculating UHC based on the selected parameters for the study

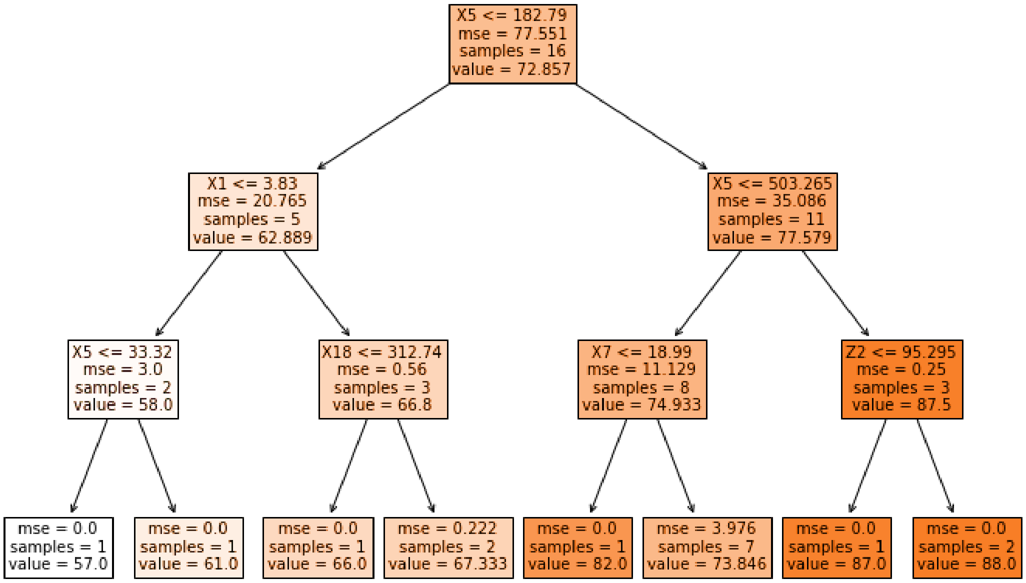

Figure A2. This decision tree illustrates the maximum of 3 level decision nodes for the classification results.

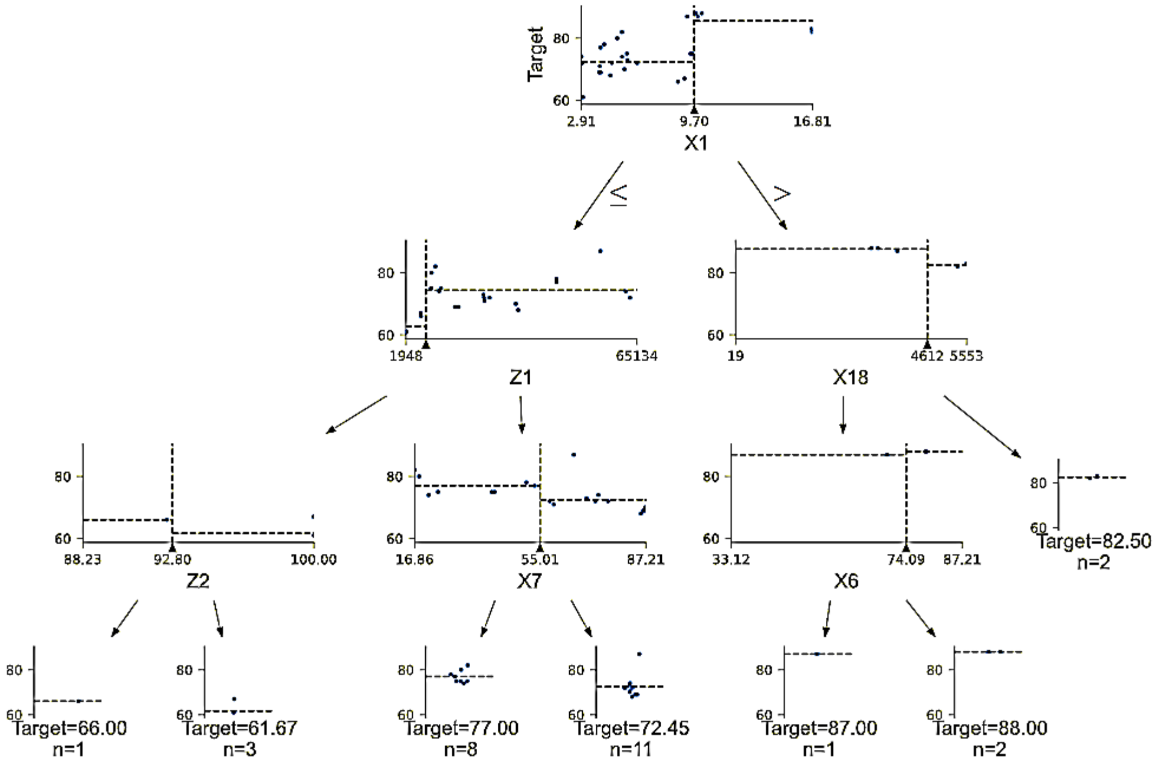

Figure A3: Calculation level overview of constructing decision trees this decision tree is one among 100 decision trees produced

The decision trees in Figures A1 and A2 summarize the supervised learning model in terms of rules at every node (i.e., at the root level and all internal nodes). The decision tree shown in A1 is one of the 100 decision trees produced in the random forest. The decision tree in figure A1 is the original version of the randomly selected tree. The same

tree by limiting the depth of visualization is illustrated in figure A2. It shows the same decision tree in figure A1 by limiting the maximum depth. Here, for the illustration purpose, we have limited the depth of the decision tree to 3 levels.

The low level – i.e., algorithmic level of the decision tree is given in figure A3. The decision boundary is shown at each level for every node in the decision tree. The decision boundaries at each level play a role in classifying the query instances. At each level, the attributes of the decision tree are inspected for various splits. The split with the minimum entropy and maximum information gain will be taken for initiating the split at any given node. The information gain and entropy are measured on the scale of bits. It is always optimal to achieve less entropy (information disorderness), and maximum information gain. Based on this principle, random forest builds numerous decision trees. The collective classification from each decision tree in the random forest will be used to classify any instance to its class. Random forest is a prevalent model for implementing multiclass classification problems. The random forest can classify the target function, both continuous and discrete.

As illustrated in figure A3, various decision boundaries are formed at different nodes of each level of the decision tree. The decision boundary is drawn based on the metrics mentioned in the previous paragraph. The internal nodes in the decision tree will facilitate the classification, and the leaf nodes of the decision tree provide the class of the instance under classification. The decision trees shown in Figures A1, A2, and A3 are constructed by considering 60% of the data related to universal health coverage. The remaining 40% of the data is used for testing and validation. It has been experimentally proved that the application of Random Forest for UHC classification gave better results (in terms of RMSE) than the OLS method. Hence, it provides a solid foundation for us to conclude that the machine learning based methods are superior compared to statistical-based methods. The predictive and classification power of the state-of-the-art techniques available in Artificial Intelligence and Machine Learning needs to be evaluated. There needs to be more work carried out in this regard. Artificial Intelligence, primarily Machine learning based methods, are promising for analysing financial data.
